# Hepatocyte nuclear factor 4α and cancer-related cell signaling pathways: a promising insight into cancer treatment

**DOI:** 10.1038/s12276-020-00551-1

**Published:** 2021-01-18

**Authors:** Duo-Duo Lv, Ling-Yun Zhou, Hong Tang

**Affiliations:** Center of Infectious Diseases, West China Hospital of Sichuan University, Chengdu, 610041 China

**Keywords:** Cancer therapy, Molecular biology

## Abstract

Hepatocyte nuclear factor 4α (HNF4α), a member of the nuclear receptor superfamily, is described as a protein that binds to the promoters of specific genes. It controls the expression of functional genes and is also involved in the regulation of numerous cellular processes. A large number of studies have demonstrated that HNF4α is involved in many human malignancies. Abnormal expression of HNF4α is emerging as a critical factor in cancer cell proliferation, apoptosis, invasion, dedifferentiation, and metastasis. In this review, we present emerging insights into the roles of HNF4α in the occurrence, progression, and treatment of cancer; reveal various mechanisms of HNF4α in cancer (e.g., the Wnt/β-catenin, nuclear factor-κB, signal transducer and activator of transcription 3, and transforming growth factor β signaling pathways); and highlight potential clinical uses of HNF4α as a biomarker and therapeutic target for cancer.

## Cancer diagnosis: Possible protein biomarker

A regulatory protein that plays complex and varied roles in different cancers may provide a diagnostic biomarker and prove useful in future treatments. Hong Tang and co-workers at Sichuan University in Chengdu, China, reviewed current understanding of the functional role of the hepatocyte nuclear factor 4α (HNF4α) protein. Abnormal expression of HNF4α appears to be involved in cell proliferation, invasion and metastasis in some cancers, but may suppress tumor growth in other cancers. HNF4α has been found to influence signaling pathways involved in cancer induction and progression in early-stage liver and colorectal cancers. It may also help build resistance to chemotherapy in gastric cancer. The presence of abnormal HNF4α expression may provide a diagnostic and prognostic biomarker for certain cancers. Further investigations are needed, but drugs that target HNF4α could be valuable in combination cancer treatments.

## Introduction

Hepatocyte nuclear factor 4α (HNF4α), a member of the nuclear receptor superfamily, is described as a protein that binds to specific DNA sequences and recruits cofactors and the transcription machinery to gene promoters^1^. As one of the key regulators, HNF4α has been widely associated with a large number of liver-specific genes in various processes, including metabolism, endoderm development and differentiation, and morphogenesis^2,3^. The expression of HNF4α in the epithelia of digestive and accessory digestive organs^4–6^ suggests that HNF4α is also important for the specific regulation of gene expression in these tissues^2,7^. Numerous studies have revealed that HNF4α may play distinct roles in different organ-specific environmental contexts^8^. These distinct roles can be attributed to different HNF4α isoforms generated by transcription from distinct promoters (P1 and P2)^7,9^.

HNF4α may regulate different signaling pathways by repressing or inducing the expression of downstream target genes to maintain normal physiological activity. Despite the promoter-driven isoforms, dysfunction of HNF4α clearly triggers the development of distinct diseases^8^. A study found that disruption of HNF4α causes embryonic lethality with defects in visceral endoderm formation^10^. Conditional knockout of HNF4α in early liver development damages the development of the hepatic epithelium and liver morphogenesis^11^. Deletion of HNF4α in the adult liver can result in impaired metabolic homeostasis^12^. In addition to these multiple known functions, HNF4α has been shown to play an important role in inflammatory processes in internal organs, and accumulating evidence suggests that it is linked to multiple types of cancer^13^.

Recently, additional emerging studies demonstrated that HNF4α is involved in the proliferation, apoptosis, invasion, and migration of cancer cells both in vitro and in vivo^4,14^. It has been found that HNF4α has either oncogenic or tumor-suppressive properties in cancer^15,16^. Aberrant expression of HNF4α is a characteristic of several types of cancer, and altered expression of HNF4α is strongly associated with the clinical outcome. Moreover, HNF4α may serve as a novel diagnostic and prognostic biomarker and an effective target for cancer therapy. However, the regulation of HNF4α in the extracellular and intracellular signaling pathways of tumor pathophysiology is relatively complex, and the underlying tumorigenic or tumor-suppressive functions and potential clinical value of HNF4α remain elusive. In this review, we focus on the emerging functional role of HNF4α in a variety of cancers and on the molecular mechanism of HNF4α in the regulation of tumor progression, and we discuss the potential therapeutic uses of HNF4α in cancer.

## Physiological role of HNF4α

HNF4α was originally identified in rat liver extracts, binding to sites required for the transcription of transthyretin and apolipoprotein CIII^1^. Via in situ hybridization analysis, Duncan et al.^5^ found that HNF4 was also expressed in the mesonephric tubules, pancreas, stomach, and intestine and, subsequently, in the metanephric tubules of the developing kidney. Based on this expression pattern, HNF4 was considered to play a role in the earliest stages of murine postimplantation development and organogenesis. Moreover, a study revealed that HNF4α is expressed at high levels in the liver and kidney and at low levels in β cells in the small intestine, colon, and pancreas^17^.

The HNF4α gene contains 13 exons, spans >70 kb, and has multiple alternative splice variants. Several splice variants of HNF4α are generated by transcription from two alternative promoters (P1 and P2) and by two different ‘3’ splicing events^18^. It has been proposed that multiple isoforms exist in mammals and that these isoforms are thought to play different physiological roles in the development and transcriptional regulation of target genes^7^. The HNF4α isoforms driven by the different promoters exhibit tissue-specific expression patterns. Specifically, P1 promoter-driven HNF4α is expressed in the fetal and adult liver and kidneys, while P2 promoter-driven HNF4α is expressed in the fetal liver and the adult pancreas and stomach; both isoforms are expressed in the large and small intestines^19,20^. Furthermore, studies have suggested that the HNF4α isoforms have different activation properties. For instance, in the liver, the expression of HNF4α is more efficiently initiated from the P2 promoter during early liver development. However, the P1 promoter begins to be favored for transcription of the HNF4α gene during liver differentiation^21^. Subsequent research suggested that HNF4α also acts as an oncoprotein that can converge on genes coding for antiapoptotic oncogenes and cytokines and may promote the development of cancer^22^. This apparent paradox could be explained by the existence of two isoform classes produced by transcription from two different promoters.

Recently, HNF4α has been demonstrated to regulate many important physiological functions of human tissues and organs. For example, HNF4 is required for the development of the liver and can regulate liver functions by controlling the expression of numerous hepatic-specific genes associated with a number of critical metabolic pathways (e.g., glycolysis, gluconeogenesis, fatty acid metabolism, urea production, bile acid synthesis, apolipoprotein synthesis, and drug metabolism)^9^. HNF4α inactivation experiments in mice clearly demonstrated the important role of this factor in liver differentiation and morphogenesis at different stages of normal development^11,23^. During embryonic colon development and intestinal epithelial cell differentiation, HNF4α is involved in the control of pancreatic β-cell proliferation, formation of crypts, maturation of mucin-producing goblet cells, and regulation of the expression of many tissue-specific genes^24^. HNF4α is differentially expressed in the renal epithelium and can regulate the expression of kidney-specific genes^25^. Moreover, HNF4α can activate the expression of multiple genes encoding cell adhesion molecules, extracellular matrix components, cytoskeletal proteins, factors involved in cell survival and proliferation control, and several other HNFs^26^.

## Emerging insights into the roles of HNF4α in cancer

**Fig. 1:**
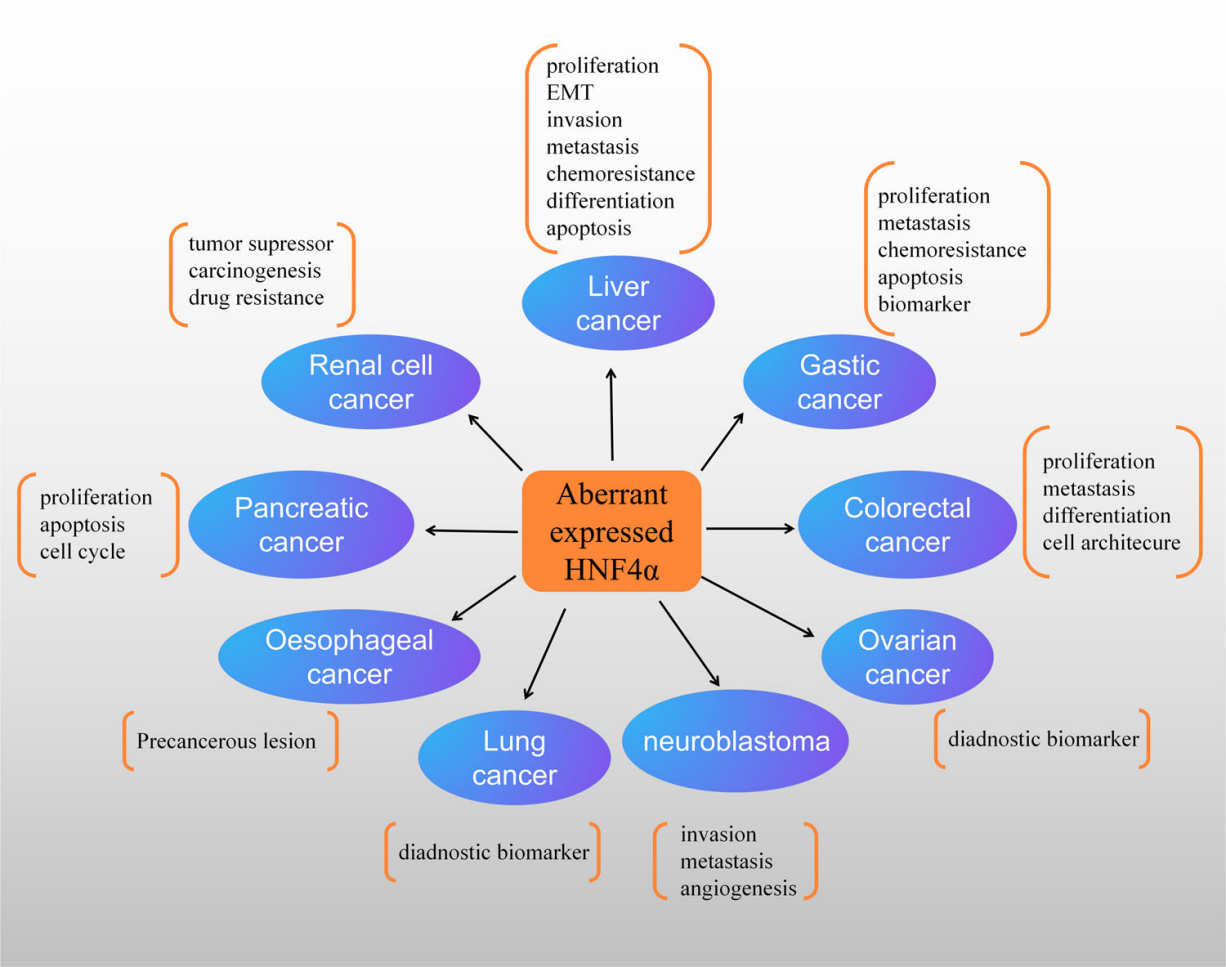
Biological functions and potential clinical applications of HNF4α in cancer. Abnormal HNF4α expression is observed in a variety of cancer types, including gastrointestinal and digestive cancers, lung cancers, urogenital cancers, and neuroblastoma, which are depicted in the above figure. In cancer, HNF4α can modulate the differentiation, proliferation, apoptosis, invasion, migration, and chemoresistance of cells and may also be used as a diagnostic biomarker.

Although there is an abundance of evidence indicating that HNF4α plays an important role in embryonic development and controlling biological functions, its role in the regulation of tumorigenesis and cancer development remains unclear. Various studies have documented that aberrant expression of HNF4α is a potential cancer-specific signature and can be correlated with clinical features in malignant tissues, indicating an important role of HNF4α in several types of cancer. Collectively, a large body of evidence shows that HNF4α is associated with the proliferation, differentiation, progression, and metastasis of cancer cells, which could be considered potential prognostic and diagnostic biomarkers during the development of cancer^2,24^. Herein, we discuss emerging insights into the roles of HNF4α in several types of cancer. Figure 1 summarizes this information.

### HNF4α in gastrointestinal cancers

#### Esophageal cancer

Barrett’s metaplasia is an important pathological condition because it is the only known morphological precursor to esophageal adenocarcinoma^27^. Colleypriest et al.^28^ confirmed that HNF4α is sufficient for the induction of a columnar-like phenotype in the adult mouse esophageal epithelium and is present in Barrett’s metaplasia in humans. This observation suggested that induction of HNF4α is a key early step in the formation of Barrett’s metaplasia and is consistent with the origin of Barrett’s metaplasia from the esophageal epithelium.

#### Gastric cancer (GC)

GC is one of the most common causes of cancer-related mortality worldwide. Recent molecular studies have begun to identify the oncogenes and tumor suppressor genes that can directly reprogram the metabolic cycle of GC cells. Notably, HNF4α is required for cell differentiation and homeostasis in the adult mouse gastric epithelium. However, its deletion causes increased proliferation and collapse of the endoplasmic reticulum and secretory architecture in chief cells in a manner dependent on the HNF4α → X-box binding protein 1 → MIST1 transcriptional sequence^29^. Recently, it has been shown that overexpression of HNF4α in GC is essential for GC proliferation in vitro and in vivo^30^. Interestingly, HNF4α acts as an oncogene in GC, and only P2-HNF4α is expressed in the stomach^20,31^. A functional study analyzing the intestinal phenotype of nonneoplastic and neoplastic gastric gland cells reported that HNF4α may be involved in the establishment and/or maintenance of the intestinal phenotype of gastric mucosa and adenocarcinoma^32^. Additionally, a study conducted by Xu et al.^14^ highlighted the role of HNF4α in sustaining oncogenic metabolism in GC cells through the regulation of IDH1. Moreover, Yubo Ma et al.^33^ demonstrated the role of HNF4α in chemoresistance in GC, suggesting that HNF4α may enhance multidrug resistance by regulating apoptosis and the expression of B-cell lymphoma 2 (BCL2). Their results also showed that overexpression of HNF4α in human GC tissue was associated with more advanced tumor stage and lymph node metastasis. Additionally, HNF4α has been proposed as a specific biomarker for distinguishing GC tissues from other types of tissues^34^. Consequently, further investigation is warranted to identify the function of HNF4α and understand the role of HNF4α in the pathological mechanism of GC and to determine its potential clinical applications.

#### Colorectal cancer (CRC)

Previously, studies have found that HNF4α is involved in the control of intestinal cell proliferation, crypt formation, mucin formation in the regulation of goblet cell maturation, and regulation of the expression of many tissue-specific genes during embryonic colon development and intestinal epithelial cell differentiation^24^. Additionally, it is a key factor in the homeostasis, cell architecture, and barrier function of the adult intestinal epithelium^35^. Recently, the role of HNF4α in intestinal cancer has been further investigated. As previously mentioned, the two promoters are expressed under unique conditions, with the large and small intestines being the only adult tissues that express both P1- and P2-HNF4α. Although some studies did not distinguish between the different HNF4α genes and protein isoforms, several recent studies showed that ectopic expression of P1-HNF4α but not P2-HNF4α reduced the tumorigenic potential of HCT116 human colon cancer cells in a mouse xenograft model^36,37^. It was also shown that P1-HNF4α exerts a differentiative effect on intestinal epithelial cells, while P2-HNF4α exerts a proliferative effect on these cells^36,38^. Chellappa et al.^37^ observed that lost or mislocalized P1-HNF4α in ~80% of Dukes stage C colon cancers was correlated with active Src. This finding revealed that Src kinase preferentially phosphorylates P1-HNF4α in vitro and in vivo at multiple residues in a complex manner, resulting in loss of function and loss of protein stability of P1-HNF4α but not P2-HNF4α. These results suggest that different HNF4α subtypes may actually play different roles in the colon. Furthermore, a study indicated that the increased transcriptional activity of HNF4α converges on antiapoptotic oncogenes and that cytokines may contribute to the development of CRC^22^. Thus, considering the unique role of HNF4α in CRC, targeting HNF4α may be a promising strategy for the treatment of CRC.

#### Liver cancer

Numerous studies have reported that the expression of HNF4α is dysregulated in hepatocellular carcinoma (HCC) and associated with the development and progression of HCC, thus providing new insight into HCC tumorigenesis^26^. Recent data suggest that HNF4α is involved in multiple mechanisms and may inhibit the proliferation of hepatocytes. Battle et al.^39^ provided evidence that HNF4α can regulate the expression of numerous proteins implicated in cell adhesion and junction assembly. As expected, loss of HNF4α led to the dedifferentiation of hepatocytes. Indeed, accompanied by a decrease in HNF4α expression, a reduction in cell–cell and cell–extracellular matrix adhesion, loss of cell polarity, an increase in telomerase activity, and inhibition of the expression of liver-specific genes occur in hepatocarcinoma^40^. In addition, it has been reported that HNF4α is a key control point for the transition to aggressive HCC (from slow-growing to rapidly proliferating HCC)^26^. For example, Yin et al.^40^ demonstrated a striking suppressive effect of HNF4α on tumorigenesis and tumor development via promotion of cancer stem cell differentiation into mature hepatocytes. This effect led to apoptosis, cell cycle arrest, and cellular senescence. The findings of another study suggested that HNF4α inhibits the proliferation of hepatocytes by downregulating the expression of oncogenes, such as c-Myc, and shed light on the mechanism underlying HNF4α-mediated inhibition of cell proliferation^3^. Previous studies have linked apoptosis signal-regulating kinase 1 (ASK1) to a variety of cellular functions and pathophysiological processes, such as proliferation, survival, and the inflammatory response^41,42^. Recently, researchers demonstrated that HNF4α can transcriptionally upregulate ASK1 by directly targeting its promoter in HCC cells. More importantly, strong suppression of ASK1 expression was correlated with decreased HNF4α levels in HCC tissues, and downregulation of ASK1 partially abrogated the HNF4α-mediated inhibition of HCC^43^. Furthermore, a recent study conducted by Saha et al. highlighted the importance of HNF4α in intrahepatic cholangiocarcinoma (IHCC). A genetically engineered mouse model of IHCC expressing mutant isocitrate dehydrogenase (IDH) showed an abnormal response to liver damage in the adult liver; this response was characterized by HNF4α silencing, impaired differentiation of hepatocytes, and markedly increased cell proliferation. These results revealed a new mechanism in which upregulation of IDH prevents differentiation of liver progenitor cells through inhibition of HNF4α^44^. Based on this evidence, it can be concluded that HNF4α may be a key regulator of liver cancer development^45^.

#### Pancreatic cancer

The expression of HNF4α has been found to be aberrant in pancreatic cancer cells. Sun et al.^46^ showed that HNF4α was upregulated in pancreatic cancer and may be an oncogene. Abrogation of HNF4α expression inhibited the proliferation of pancreatic cancer cells and induced their apoptosis, with increased expression of the cyclin-dependent protein kinase inhibitors p21 and p27. In addition, this study demonstrated that increased HNF4α expression in pancreatic adenocarcinoma was responsible for pancreatic cancer cell proliferation and promoted resistance to gemcitabine by downregulating hENT1^46^. Thus, HNF4α may serve as a prognostic marker for overall survival, and targeting HNF4α might reverse gemcitabine resistance and provide novel treatment strategies for pancreatic adenocarcinoma.

### Lung cancer

In some instances, diagnosis of invasive mucinous adenocarcinoma of the lung from a biopsy specimen is difficult because of its minimal nuclear atypia and sparse tumor cells. However, HNF4 (a positive marker) could be useful for identifying invasive mucinous lung adenocarcinoma cells^47^. Furthermore, aiming to clarify the development of a normal counterpart and precancerous lesion of non-terminal respiratory unit (TRU) origin in lung adenocarcinomas, Koji Okudela et al. found that the expression of HNF4α was similar between bronchiolar metaplastic lesions and terminal bronchioles and that some of the metaplastic lesions exhibited an unequivocally higher frequency and expression level of HNF4α comparable to that observed in non-TRU lung adenocarcinomas^48^. Therefore, bronchiolar metaplastic lesions strongly expressing HNF4α are considered precancerous lesions of non-TRU lung adenocarcinomas.

### HNF4α in urogenital cancers

#### Renal cell carcinoma (RCC)

Sel et al.^49^ was the first to describe altered HNF4α expression in human RCC by showing its increased expression and DNA binding activity. Subsequently, Lucas et al.^25^ showed based on its downregulation in RCC that HNF4α played a role as a tumor suppressor. A study revealed that the mRNA levels of *HNF4α* in RCC were downregulated by 4.7-fold^50^. Notably, many studies found a strong correlation between the expression of HNF4α and E-cadherin in high-grade RCC, which suggests that the regulation of E-cadherin by HNF4α may be closely associated with the malignancy of RCC^51^. These results revealed that HNF4α was downregulated in RCC and that its downregulation was associated with a poor prognosis in patients with RCC. Moreover, inactivation of HNF4α transcription showed that increased expression and DNA binding activity of HNF4α contribute to carcinogenesis and drug resistance in clear-cell RCC^52^. Thus, restoration of HNF4α could render RCC cells more sensitive to chemotherapy. For example, Hagos et al.^53^ showed that HNF4α increased the expression of organic cation and anion transporters in RCCNG1 cells, thereby increasing the chemosensitivity of tumor cells to oxaliplatin and fluorouracil.

#### Ovarian cancer

HNF4α is expressed in several endodermal tissues. A recent study used a cytological approach to determine that cancer cells in ascites samples from patients with mucinous ovarian adenocarcinoma were HNF4α-positive and that tumor cells in ascites samples from patients with other types of ovarian cancer were HNF4α-negative^54^. Therefore, HNF4α was revealed to be a useful marker for the histological and cytological diagnosis of ovarian mucinous tumors.

### Neuroblastoma

Neuroblastoma is an extracranial solid tumor that occurs in children and arises from sympathetic neurons via a complex mechanism^55^. A recent analysis of clinical neuroblastoma tissue samples revealed that HNF4α promoted the invasion, metastasis, and angiogenesis of neuroblastoma cells by targeting matrix metalloproteinase 14^56^. Moreover, Li and Chen^57^ reported that the overexpression of miR-34a inhibits the proliferation, migration, and invasion of human neuroblastoma SH-SY5Y cells by targeting HNF4α. Additionally, Defeng Deng et al.^58^ reported that the long noncoding RNA small nucleolar RNA host gene 16 plays an oncogenic role though the miR-542-3p/HNF4α axis via the RAS/RAF/MEK/ERK signaling pathway to induce neuroblastoma growth. These results clarify the functional importance of HNF4α in neuroblastoma progression.

## Signaling pathways of HNF4α in tumor regulation

**Fig. 2:**
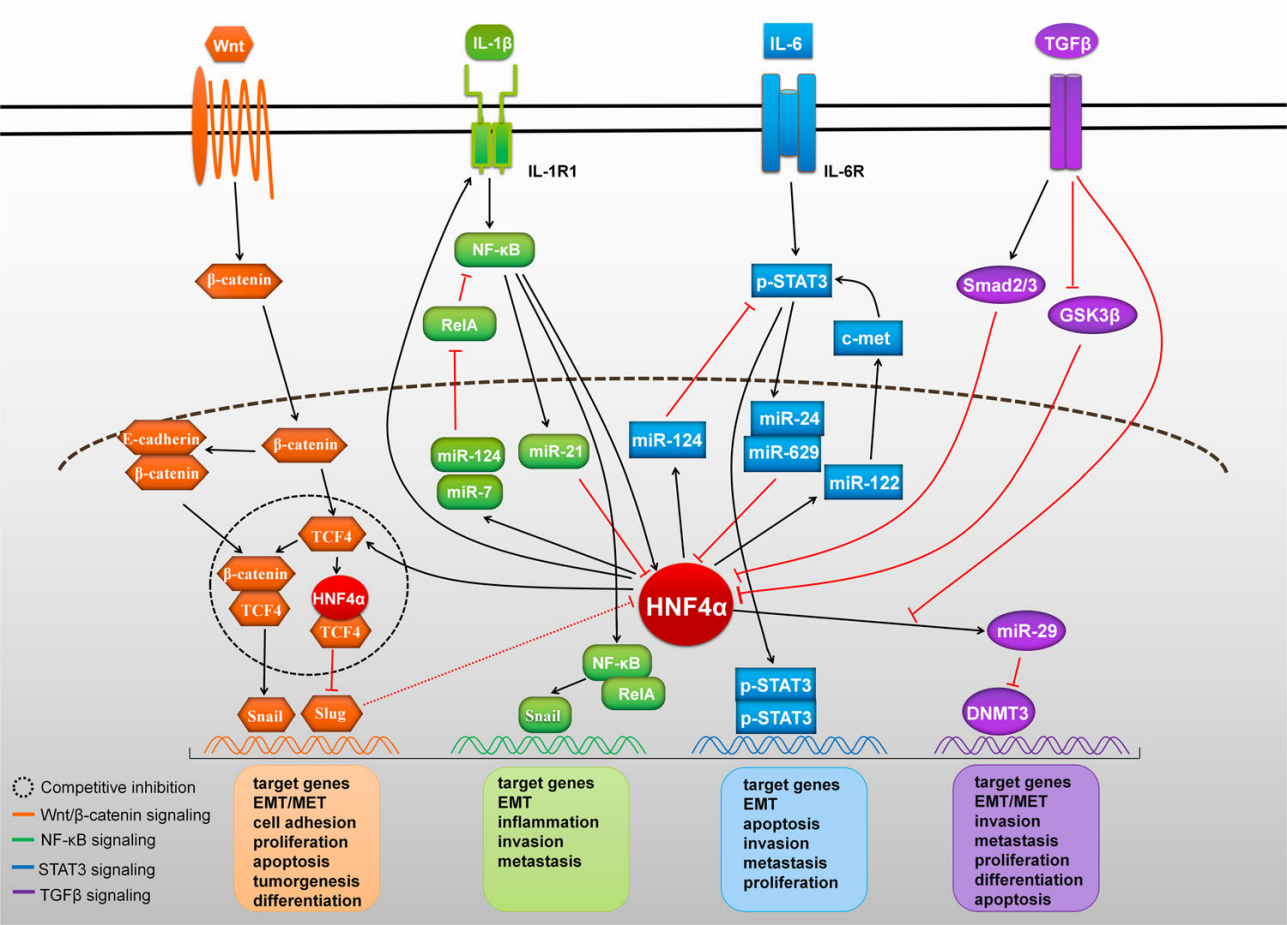
Role of aberrant HNF4α-related cell signaling pathways in cancer. HNF4α in cancer cells mainly acts through the Wnt/β-catenin, NF-κB, STAT3, and TGFβ signaling pathways to increase cell migration and invasion and decrease apoptosis. High expression of HNF4α regulates target genes by inhibiting the Wnt/β-catenin, STAT3 and TGFβ signaling pathways. After induction by stimuli, high HNF4α expression can activate the NF-κB signaling pathway to promote tumor progression or inhibit the NF-κB signaling pathway to downregulate target genes. Aberrant HNF4α-related Wnt/β-catenin, NF-κB, STAT3 and TGFβ signaling pathway activity is involved throughout the EMT process.

In cancer, numerous signaling pathways may have diverse functions and be defined as an interconnected network modulating complex phenomena through a molecular mechanism. Although the major physiological function of signaling pathways is to maintain homeostasis, signaling in normal and oncogenic cells is significantly different. HNF4α is associated with many signaling pathways that play an important role in tumor transformation, metastasis, inhibition of apoptosis, and promotion of proliferation. Recently, it has been shown that HNF4α is involved in abnormal activation of one or more signaling pathways (such as the nuclear factor-κB (NF-κB) pathway, Wnt/β-catenin pathway, and STAT pathway), playing a pivotal role in the occurrence and progression of cancer (Fig. 2).

### Wnt/β-catenin pathway

Dysregulation of the Wnt/β-catenin signaling pathway is involved in various types of cancer. Researchers previously reported that overexpression of HNF4α can suppress tumor development through downregulation of the Wnt/β-catenin signaling pathway^59^. Overexpression of HNF4α in cells with a dedifferentiated malignant phenotype restored the cells to an epithelial-like phenotype, indicating that HNF4α is a regulator of epithelial–mesenchymal transition (EMT). It is well established that EMT is a complex multistep biological process orchestrated by a variety of EMT-inducing transcription factors. This process induces the transdifferentiation of epithelial-like cells into mesenchymal-like cells and facilitates their invasion and migration into blood vessels and lymphatic vessels, thereby participating in the metastasis of a variety of cancers^60,61^. Notably, inhibition of the Wnt/β-catenin pathway may downregulate EMT-related markers and decrease cell proliferation and migration^62^. Meng Yang et al.^63^ found that overexpression of HNF4α completely abolished the Wnt/β-catenin signaling-induced EMT phenotype. In particular, HNF4α inhibits the activation of β-catenin, which is upstream of SNAIL/SLUG and binds competitively to transcription factor 4 (TCF4) in the nucleus^63^. Conversely, SNAIL inhibits the expression of HNF4α^64^, thereby forming a β-catenin-SNAIL/SLUG-HNF4α negative feedback circuit. HNF4α also recruits transcriptional repressors to the promoters of Wnt target genes, further inhibiting the transcription of Wnt/β-catenin signaling pathway target genes (e.g., SLUG and AXIN2)^63^. In addition, HNF4α can relocate β-catenin from the nucleus to the cell membrane, participate in adhesion junctions between epithelial cells, strengthen the epithelial phenotype of cells, reverse the EMT phenotype, and promote the activation of mesenchymal–epithelial transition (MET)^65^. In addition, HNF4α can directly inhibit the expression of EMT regulatory factors (SNAIL and SLUG) and transform the hepatocyte EMT phenotype into a MET phenotype, thereby inhibiting the progression of cancer^66^. Thus, the double-negative feedback loop formed between Wnt/β-catenin signaling and HNF4α is involved in the regulation of cancer progression.

### NF-κB pathway

Early studies showed that NF-κB is a central factor in inflammation, cell differentiation and proliferation, and cell death and can be activated by a large variety of stimuli^67^. Recently, NF-κB signaling has been shown to be activated in cancer stem cells, promoting a proinflammatory environment, inhibiting apoptosis, and stimulating cell proliferation^68^. As expected, HNF4α is involved in the regulation of NF-κB signaling in cancer progression. HNF4α stimulates the expression of interleukin 1 receptor type 1 (IL1R1) and then amplifies the inflammatory response evoked by its ligand interleukin 1β (IL1β). IL1β/IL1R1 activates NF-κB signaling, thereby increasing the expression of HNF4α and forming a feedback loop that sustains activation of the NF-κB pathway and drives inflammation toward cancer^69^. In addition, studies have suggested that microRNAs and HNF4α may cooperate to tune gene expression in distinct biological and pathological processes^70^. Ning et al.^71^ found that HNF4α directly upregulated the expression of miR-7 and miR-124 in carcinoma cells and downregulated that of the NF-κB subunit RELA, thereby inhibiting the induction of carcinoma via the NF-κB signaling pathway. Moreover, NF-κB was found to upregulate the expression of miR-21 and inhibit that of HNF4α, thereby forming an HNF4α-NF-κB negative feedback regulatory loop to regulate the course of cancer. Furthermore, NF-κB promotes and maintains an invasive phenotype of cells and functions as an essential mediator of EMT^72^. For example, scholars directly introduced the SNAIL gene into a mouse liver cell line and found that it induced EMT accompanied by a decline in the expression of HNF4α. After exogenous introduction of the HNF4α gene, SNAIL-induced EMT was blocked in liver cells^64,73^. Therefore, in cooperation with microRNAs, HNF4α inhibits the activation and degradation of SNAIL via NF-κB by downregulating the expression of RELA, thereby blocking the EMT process in tumor cells and alleviating or reversing the pathology of cancer.

### HNF4α-signal transducer and activator of transcription 3 (HNF4α-STAT3) pathway

The link between STAT family proteins and carcinoma in humans is well demonstrated, and constitutively activated STAT3 is crucial for carcinogenesis^74^. STAT3 is considered an oncogene and is highly expressed in a variety of tumor tissues and cells^75^. Sustained activation of STAT3 can cause abnormal proliferation and malignant transformation of tumor cells, enhance the antiapoptotic ability of tumors, and promote tumor invasion, metastasis, and angioplasty^76^. Moreover, the transcriptional program activated by phosphorylated STAT3 in tumors results in the formation of rapidly growing lesions that are highly metastatic^77^. In addition, it has been suggested that phosphorylated STAT3 is positively associated with the expression of the transcription factor TWIST, which is involved in EMT induction, and is negatively correlated with the expression of the epithelial cell marker E-cadherin^78^. E-cadherin is an important factor in invasion and metastasis. Loss of E-cadherin expression stimulates the transformation of cells into a more invasive and less differentiated state through the EMT process^79^. Therefore, activated STAT3 can promote the invasion and metastasis of cancer cells by mediating the EMT process. Hatziapostolou et al.^80^ found that HNF4α inhibits the activation of STAT3 by directly upregulating the expression of miR-124, thereby blocking the activation of STAT3. They also reported that STAT3 is inhibited by upregulation of miR-24 and miR-629 expression. Expression of HNF4α forms an HNF4α-STAT3 feedback regulatory loop that regulates the course of carcinoma. Moreover, HNF4α can cause dysregulation of miR-122 to promote the induction of c-Met and activate the phosphorylation of STAT3, contributing to cancer aggressiveness^81^. Therefore, HNF4α can alleviate or reverse tumor lesions by blocking the activation of the STAT3 signal transduction pathway and inhibiting the invasion and metastasis of cancer cells.

### Transforming growth factor β (TGFβ) signaling

The TGFβ signaling pathway plays important roles in regulating various biological processes, including cell growth, apoptosis, migration, invasion, etc^82^. Previous reports have suggested that TGFβ signaling plays a dual, opposite role in carcinogenesis. In normal and premalignant cells, it can act as a tumor suppressor. In contrast, during the malignant phases of cancer progression, the TGFβ signaling pathway triggers tumor-promoting effects, particularly by driving EMT. This event enhances tumor cell migration, invasion, and metastasis to distant organs and ultimately increases resistance to apoptotic stimuli and chemotherapy^82,83^. Interestingly, during postnatal liver development in mice, HNF4α and TGFβ are among the first three upstream regulators of gene expression involved^84^. TGFβ plays a leading role in inhibiting the function of HNF4α through transcriptional repression and posttranslational modification of HNF4α^85,86^. TGFβ inhibits the activity of HNF4α by targeting this protein for proteasomal degradation in tumor cells^85^. The presence of TGFβ impairs the efficiency of HNF4α as a tumor suppressor. Moreover, TGFβ induces posttranslational modifications of HNF4α, which result in early loss of HNF4α DNA binding activity toward the target gene promoter^86^. The results of that study also showed that chemical inhibition of glycogen synthase kinase 3β (GSK3β) leads to impairment of HNF4α binding to DNA. Hence, GSK3β kinase is one of the TGFβ targets that mediates the inactivation of HNF4α^86^. In addition, HNF4α exerts epigenetic control of the EMT/MET state in differentiated hepatocytes through miR-29-mediated downregulation of DNA methyltransferases (DNMTs)^87,88^. The degree of miR-29 downregulation and DNMT upregulation is associated with TGFβ-induced EMT and the aggressiveness of cancer^89^. It was further demonstrated that persistent high levels of DNMT maintain DNA methylation, inducing epigenetic changes and participating in EMT and cancer^90,91^. These results reveal that epigenetic regulation of genes by HNF4α and TGFβ can be seen as two unique EMT mechanisms in carcinogenesis. Taken together, these results indicate that there is extensive interaction between HNF4*α* and TGFβ during cancer progression.

## Therapeutic insights into HNF4α

Recent evidence suggests that HNF4α is involved in the proliferation of a variety of cell types throughout the body and can be used as a potential therapeutic target. Nuclear receptors are major therapeutic targets in several metabolic disorders and cancer. This function is largely attributed to their hydrophobic ligand-binding pockets, which are natural targets of small molecules and help regulate the recruitment of coregulators^13^. As a member of the nuclear receptor superfamily of transcription factors, HNF4α has been reported to possess enormous potential as a clinical therapeutic target in several types of cancer. Yuan et al.^92^ was the first to demonstrate that HNF4α binds reversibly to the essential fatty acid linoleic acid in mammalian cell culture and mouse liver. This finding suggests the possibility of HNF4α as a drug target. Additional therapeutic drugs can be designed based on the characteristics of HNF4α, especially for the treatment of cancer. In this context, the pivotal role of HNF4α as a tumor suppressor indicates the design of promising strategies for the treatment of HCC based on the restoration of HNF4α expression and function. There is substantial evidence supporting HNF4α as a “drug” for the treatment of HCC. Marchetti et al.^93^ recently reported the use of members of the liver-enriched transcription factor family, particularly HNF4α, as a tool for gene therapy against HCC. As a master regulator of EMT/MET, HNF4α dynamically restores the differentiation of hepatocytes, induces MET in HCC cells, and controls the epigenetic modification state of differentiated hepatocytes via downregulation of DNA methyltransferases^88^. TGFβ overrides the tumor-suppressive activity of HNF4α through the inactivation of GSK3β. Future gene therapies against HCC can be developed based on the inhibition of HNF4α by TGFβ^86^. Moreover, from previous research, we have learned that the phosphorylation of paxillin at Tyr118 and autophosphorylation of Src are vital biomarkers of dasatinib activity in tumors for assessing the efficiency of Src activity and tumor growth inhibition^94^. Current Src inhibitors used to treat CRC include dasatinib, AZD-0530, and SKI-606, which are in phase I or phase II clinical trials^13^. Notably, the tyrosine kinase c-Src markedly inhibits the activity of P1-HNF4α but not that of products of P2-HNF4α via selective phosphorylation of P1-HNF4α at tyrosine 23 (Tyr 23) and tyrosine 286 (Tyr 286). This finding indicates that phosphorylation of Tyr 23 and Tyr 286 may be another predictive biomarker for the therapeutic efficacy of Src inhibitors in CRC^37^. Conversely, previous studies have shown that HNF4α is specifically overexpressed in GC and is functionally required for the development of GC. Xu et al.^14^ found that the function of HNF4α in maintaining the oncogenic metabolism of GC cells can be achieved by regulating IDH1. Therefore, the results of therapeutic studies based on HNF4α indicate that drug design and development can be performed based on the regulation of HNF4α in different tumors. In conclusion, HNF4α has long been recognized as an important regulator of differentiation and is currently associated with cancer. The link between HNF4α and various cancers can be used to predict the susceptibility of tumors to treatment. While investigations into therapeutic methods based on HNF4α are currently in early stages, more therapeutic achievements could be attained in the future with an increased understanding of the mechanisms and functions of HNF4α in cancer. Similarly, the role of different HNF4α isoforms in cancer is worthy of further study. Therapeutic drugs for different cancers are shown in Table 1.

**Table 1:** A comprehensive list of therapeutic drugs targeting HNF4α activity in cancer.

	Cancer type	Mechanism of drug action	References
Gene therapy (delivery of LETF)	Liver cancer	Induces MET and epithelial/hepatic differentiation and blocks EMT carcinogenesis and metastasis	^93^
Oroxylin A	Liver cancer	Activates the PKM1/HNF4α pathway	^95^
5-Aza-Cd	Liver cancer	Induces PPARγ/RXRα and restores miR-122 expression	^96^
BI6015	Gastric cancer	Suppresses the Wnt and Notch embryonic signaling pathways	^97^
Berberine	Gastric cancer	Is involved in the AMPK-HNF4α-WNT5A signaling pathway	^98^
HDAC inhibitors	Colon carcinoma	Downregulates MUC4	^99^
Dasatinib, AZD-0530 and SKI-606	Colorectal cancer	Increases P1-HNF4α protein levels and suppresses colon cancer progression	^13^
Oxaliplatin and 5-FU	Renal cell carcinoma	Overexpression of HNF4α induces chemosensitivity to oxaliplatin and 5-FU mediated by OCT1 and CNT3	^53^
Apicidin (histone deacetylase inhibitor)	Pancreatic cancer	Reduces the expression of MUC4 and its transcription factor HNF4α	^100^
miR-34a	Neuroblastoma	Targets HNF4α to inhibit proliferation, migration and invasion	^57^

## Conclusion and future perspectives

In this review, we summarized the molecular mechanisms associated with HNF4α that regulate multiple processes in cancer. In particular, HNF4α is abnormally expressed in a cancer-specific manner in various types of tumors and has opposite functions in tumor inhibition and promotion. Overexpression of HNF4α in different types of tumor cells (e.g., HCC, CRC, and RCC cells) is recognized as a major antitumor factor in suppressing EMT, disease progression, and metastasis; however, it exerts an opposite effect in GC, lung cancer, pancreatic cancer, and neuroblastoma. Therefore, further understanding of the regulatory mechanisms of HNF4α in different cell types in patients with cancer has the potential to improve the antitumor efficacy of targeting HNF4α and/or to overcome chemoresistance. Although numerous studies have demonstrated that HNF4α is dysregulated in cancers and may serve as a novel diagnostic biomarker and therapeutic target in cancers, clinical application of HNF4α remains challenging. Moreover, drugs that target HNF4α (identified in mechanistic studies) have the potential to increase these benefits when used in combination with other chemotherapeutic drugs to treat tumors.

## References

[1] Sladek FM, Zhong WM, Lai E, Darnell JE (1990). Liver-enriched transcription factor HNF-4 is a novel member of the steroid hormone receptor superfamily. Genes Dev..

[2] Yamagata K (1996). Mutations in the hepatocyte nuclear factor-4alpha gene in maturity-onset diabetes of the young (MODY1). Nature.

[3] Walesky C (2013). Hepatocyte nuclear factor 4 alpha deletion promotes diethylnitrosamine-induced hepatocellular carcinoma in rodents. Hepatology.

[4] Babeu JP, Darsigny M, Lussier CR, Boudreau F (2009). Hepatocyte nuclear factor 4alpha contributes to an intestinal epithelial phenotype in vitro and plays a partial role in mouse intestinal epithelium differentiation. Am. J. Physiol. Gastrointest. Liver Physiol..

[5] Duncan SA (1994). Expression of transcription factor HNF-4 in the extraembryonic endoderm, gut, and nephrogenic tissue of the developing mouse embryo: HNF-4 is a marker for primary endoderm in the implanting blastocyst. Proc. Natl Acad. Sci. USA.

[6] Taraviras S, Monaghan AP, Schutz G, Kelsey G (1994). Characterization of the mouse HNF-4 gene and its expression during mouse embryogenesis. Mechanisms Dev..

[7] Jiang S (2003). Expression and localization of P1 promoter-driven hepatocyte nuclear factor-4alpha (HNF4alpha) isoforms in human and rats. Nucl. receptor.

[8] Babeu JP, Boudreau F (2014). Hepatocyte nuclear factor 4-alpha involvement in liver and intestinal inflammatory networks. World J. Gastroenterol..

[9] Lu H (2016). Crosstalk of HNF4alpha with extracellular and intracellular signaling pathways in the regulation of hepatic metabolism of drugs and lipids. Acta Pharm. Sin. B.

[10] Chen WS (1994). Disruption of the HNF-4 gene, expressed in visceral endoderm, leads to cell death in embryonic ectoderm and impaired gastrulation of mouse embryos. Genes Dev..

[11] Parviz F (2003). Hepatocyte nuclear factor 4alpha controls the development of a hepatic epithelium and liver morphogenesis. Nat. Genet..

[12] Hayhurst GP, Lee YH, Lambert G, Ward JM, Gonzalez FJ (2001). Hepatocyte nuclear factor 4alpha (nuclear receptor 2A1) is essential for maintenance of hepatic gene expression and lipid homeostasis. Mol. Cell. Biol..

[13] Chellappa K, Robertson GR, Sladek FM (2012). HNF4alpha: a new biomarker in colon cancer?. Biomark. Med..

[14] Xu, C. et al. HNF4alpha pathway mapping identifies wild-type IDH1 as a targetable metabolic node in gastric cancer. *Gut*10.1136/gutjnl-2018-318025 (2019). 10.1136/gutjnl-2018-318025 31068366

[15] Chellappa, K. et al. Opposing roles of nuclear receptor HNF4alpha isoforms in colitis and colitis-associated colon cancer. *Elife*10.7554/eLife.10903 (2016). 10.7554/eLife.10903 PMC4907689 27166517

[16] Tsai PH (2019). Dual delivery of HNF4alpha and cisplatin by mesoporous silica nanoparticles inhibits cancer pluripotency and tumorigenicity in hepatoma-derived CD133-expressing stem cells. ACS Appl Mater. Interfaces.

[17] Walesky C, Apte U (2015). Role of hepatocyte nuclear factor 4alpha (HNF4alpha) in cell proliferation and cancer. Gene Expr..

[18] Yusuf D (2012). The transcription factor encyclopedia. Genome Biol..

[19] Tanaka T (2006). Dysregulated expression of P1 and P2 promoter-driven hepatocyte nuclear factor-4alpha in the pathogenesis of human cancer. J. Pathol..

[20] Dean S, Tang JI, Seckl JR, Nyirenda MJ (2010). Developmental and tissue-specific regulation of hepatocyte nuclear factor 4-alpha (HNF4-alpha) isoforms in rodents. Gene Expr..

[21] Torres-Padilla ME, Fougere-Deschatrette C, Weiss MC (2001). Expression of HNF4alpha isoforms in mouse liver development is regulated by sequential promoter usage and constitutive 3’ end splicing. Mechanisms Dev..

[22] Schwartz B (2009). Inhibition of colorectal cancer by targeting hepatocyte nuclear factor-4alpha. Int. J. Cancer.

[23] Li J, Ning G, Duncan SA (2000). Mammalian hepatocyte differentiation requires the transcription factor HNF-4alpha. Genes Dev..

[24] Garrison WD (2006). Hepatocyte nuclear factor 4alpha is essential for embryonic development of the mouse colon. Gastroenterology.

[25] Lucas B (2005). HNF4alpha reduces proliferation of kidney cells and affects genes deregulated in renal cell carcinoma. Oncogene.

[26] Lazarevich NL (2010). Deregulation of hepatocyte nuclear factor 4 (HNF4)as a marker of epithelial tumors progression. Exp. Oncol..

[27] Fitzgerald RC (2006). Molecular basis of Barrett’s oesophagus and oesophageal adenocarcinoma. Gut.

[28] Colleypriest BJ (2017). Hnf4alpha is a key gene that can generate columnar metaplasia in oesophageal epithelium. Differentiation.

[29] Moore BD, Khurana SS, Huh WJ, Mills JC (2016). Hepatocyte nuclear factor 4alpha is required for cell differentiation and homeostasis in the adult mouse gastric epithelium. Am. J. Physiol. Gastrointest. Liver Physiol..

[30] Chia NY (2015). Regulatory crosstalk between lineage-survival oncogenes KLF5, GATA4 and GATA6 cooperatively promotes gastric cancer development. Gut.

[31] Chang HR (2016). HNF4alpha is a therapeutic target that links AMPK to WNT signalling in early-stage gastric cancer. Gut.

[32] Kojima K (2006). The expression of hepatocyte nuclear factor-4alpha, a developmental regulator of visceral endoderm, correlates with the intestinal phenotype of gastric adenocarcinomas. Pathology.

[33] Ma Y, Wei X, Wu Z (2017). HNF-4alpha promotes multidrug resistance of gastric cancer cells through the modulation of cell apoptosis. Oncol. Lett..

[34] van der Post RS (2014). HNF4A immunohistochemistry facilitates distinction between primary and metastatic breast and gastric carcinoma. Virchows Arch..

[35] Cattin AL (2009). Hepatocyte nuclear factor 4alpha, a key factor for homeostasis, cell architecture, and barrier function of the adult intestinal epithelium. Mol. Cell. Biol..

[36] Vuong LM (2015). Differential effects of hepatocyte nuclear factor 4alpha isoforms on tumor growth and t-cell factor 4/AP-1 interactions in human colorectal Cancer cells. Mol. Cell. Biol..

[37] Chellappa K (2012). Src tyrosine kinase phosphorylation of nuclear receptor HNF4alpha correlates with isoform-specific loss of HNF4alpha in human colon cancer. Proc. Natl Acad. Sci. USA.

[38] Babeu, J. P., Jones, C., Geha, S., Carrier, J. C. & Boudreau, F. P1 promoter-driven HNF4alpha isoforms are specifically repressed by beta-catenin signaling in colorectal cancer cells. *J. Cell Sci.*10.1242/jcs.214734 (2018). 10.1242/jcs.214734 29898915

[39] Battle MA (2006). Hepatocyte nuclear factor 4alpha orchestrates expression of cell adhesion proteins during the epithelial transformation of the developing liver. Proc. Natl Acad. Sci. USA.

[40] Yin C (2008). Differentiation therapy of hepatocellular carcinoma in mice with recombinant adenovirus carrying hepatocyte nuclear factor-4alpha gene. Hepatology.

[41] Ichijo H (1997). Induction of apoptosis by ASK1, a mammalian MAPKKK that activates SAPK/JNK and p38 signaling pathways. Science.

[42] Matsuzawa A (2005). ROS-dependent activation of the TRAF6-ASK1-p38 pathway is selectively required for TLR4-mediated innate immunity. Nat. Immunol..

[43] Jiang CF (2016). Apoptosis signal-regulating kinase 1 mediates the inhibitory effect of hepatocyte nuclear factor-4alpha on hepatocellular carcinoma. Oncotarget.

[44] Saha SK (2014). Mutant IDH inhibits HNF-4alpha to block hepatocyte differentiation and promote biliary cancer. Nature.

[45] Yan H (2018). Identification of potential transcription factors, long noncoding RNAs, and microRNAs associated with hepatocellular carcinoma. J. Cancer Res. Ther..

[46] Sun Q (2019). Role of hepatocyte nuclear factor 4 alpha in cell proliferation and gemcitabine resistance in pancreatic adenocarcinoma. Cancer Cell Int..

[47] Sugano M (2013). HNF4alpha as a marker for invasive mucinous adenocarcinoma of the lung. Am. J. Surg. Pathol..

[48] Okudela K (2019). A subpopulation of airway epithelial cells that express hepatocyte nuclear factor 4alpha - its implication in the development of non-terminal respiratory unit-type lung adenocarcinoma. Histol. Histopathol..

[49] Sel S, Ebert T, Ryffel GU, Drewes T (1996). Human renal cell carcinogenesis is accompanied by a coordinate loss of the tissue specific transcription factors HNF4 alpha and HNF1 alpha. Cancer Lett..

[50] Wirsing A, Senkel S, Klein-Hitpass L, Ryffel GU (2011). A systematic analysis of the 3’UTR of HNF4A mRNA reveals an interplay of regulatory elements including miRNA target sites. PLoS ONE.

[51] Gao Y (2019). HNF4alpha downregulation promotes tumor migration and invasion by regulating Ecadherin in renal cell carcinoma. Oncol. Rep..

[52] Gao YH (2017). VHL deficiency augments anthracycline sensitivity of clear cell renal cell carcinomas by down-regulating ALDH2. Nat. Commun..

[53] Hagos Y (2014). HNF4alpha induced chemosensitivity to oxaliplatin and 5-FU mediated by OCT1 and CNT3 in renal cell carcinoma. J. Pharm. Sci..

[54] Sugai M (2008). Expression of hepatocyte nuclear factor 4 alpha in primary ovarian mucinous tumors. Pathol. Int..

[55] Al-Shammari, N. F., Redha, E. & Al Hajeri, M. H. Cervical neonatal neuroblastoma with recurrent SVT. *Gulf J. Oncol.* 45–57 (2009). 20194085

[56] Xiang X (2015). Hepatocyte nuclear factor 4 alpha promotes the invasion, metastasis and angiogenesis of neuroblastoma cells via targeting matrix metalloproteinase 14. Cancer Lett..

[57] Li Z, Chen H (2019). miR-34a inhibits proliferation, migration and invasion of paediatric neuroblastoma cells via targeting HNF4alpha. Artif. Cells Nanomed. Biotechnol..

[58] Deng, D., Yang, S. & Wang, X. Long non-coding RNA SNHG16 regulates cell behaviors through miR-542-3p/HNF4alpha axis via RAS/RAF/MEK/ERK signaling pathway in pediatric neuroblastoma cells. *Biosci. Rep.*10.1042/BSR20200723 (2020). 10.1042/BSR20200723 PMC7251324 32412051

[59] Wu N (2016). Overexpression of hepatocyte nuclear factor 4alpha in human mesenchymal stem cells suppresses hepatocellular carcinoma development through Wnt/beta-catenin signaling pathway downregulation. Cancer Biol. Ther..

[60] Thiery JP, Acloque H, Huang RY, Nieto MA (2009). Epithelial-mesenchymal transitions in development and disease. Cell.

[61] Nieto MA (2009). Epithelial-mesenchymal transitions in development and disease: old views and new perspectives. Int. J. Dev. Biol..

[62] Huang Q (2017). Tg737 regulates epithelial-mesenchymal transition and cancer stem cell properties via a negative feedback circuit between Snail and HNF4alpha during liver stem cell malignant transformation. Cancer Lett..

[63] Yang M (2013). A double-negative feedback loop between Wnt-beta-catenin signaling and HNF4alpha regulates epithelial-mesenchymal transition in hepatocellular carcinoma. J. Cell Sci..

[64] Cicchini C (2006). Snail controls differentiation of hepatocytes by repressing HNF4alpha expression. J. Cell Physiol..

[65] Ning BF (2010). Hepatocyte nuclear factor 4 alpha suppresses the development of hepatocellular carcinoma. Cancer Res.

[66] Yao D, Peng S, Dai C (2013). The role of hepatocyte nuclear factor 4alpha in metastatic tumor formation of hepatocellular carcinoma and its close relationship with the mesenchymal-epithelial transition markers. BMC Cancer.

[67] Hoesel B, Schmid JA (2013). The complexity of NF-kappaB signaling in inflammation and cancer. Mol. Cancer.

[68] Chefetz I, Holmberg JC, Alvero AB, Visintin I, Mor G (2011). Inhibition of Aurora-A kinase induces cell cycle arrest in epithelial ovarian cancer stem cells by affecting NFkB pathway. Cell Cycle.

[69] Ma L (2016). Mutual amplification of HNF4alpha and IL-1R1 composes an inflammatory circuit in Helicobacter pylori associated gastric carcinogenesis. Oncotarget.

[70] Morimoto A (2017). An HNF4alpha-microRNA-194/192 signaling axis maintains hepatic cell function. J. Biol. Chem..

[71] Ning BF (2014). Hepatocyte nuclear factor 4alpha-nuclear factor-kappaB feedback circuit modulates liver cancer progression. Hepatology.

[72] Huang T, Chen Z, Fang L (2013). Curcumin inhibits LPS-induced EMT through downregulation of NF-kappaB-Snail signaling in breast cancer cells. Oncol. Rep..

[73] Tsubaki M (2013). Activation of NF-kappaB by the RANKL/RANK system up-regulates snail and twist expressions and induces epithelial-to-mesenchymal transition in mammary tumor cell lines. J. Exp. Clin. Cancer Res..

[74] Grandis JR (1998). Requirement of Stat3 but not Stat1 activation for epidermal growth factor receptor- mediated cell growth In vitro. J. Clin. Invest.

[75] Avalle L, Pensa S, Regis G, Novelli F, Poli V (2012). STAT1 and STAT3 in tumorigenesis: a matter of balance. JAKSTAT.

[76] Loh CY (2019). Signal transducer and activator of transcription (STATs) proteins in cancer and inflammation: functions and therapeutic implication. Front. Oncol..

[77] Haricharan S, Li Y (2014). STAT signaling in mammary gland differentiation, cell survival and tumorigenesis. Mol. Cell Endocrinol..

[78] Yang J (2004). Twist, a master regulator of morphogenesis, plays an essential role in tumor metastasis. Cell.

[79] Bure, I. V., Nemtsova, M. V. & Zaletaev, D. V. Roles of E-cadherin and noncoding RNAs in the epithelial-mesenchymal transition and progression in gastric cancer. *Int. J. Mol. Sci*. 10.3390/ijms20122870 (2019). 10.3390/ijms20122870 PMC6627057 31212809

[80] Hatziapostolou M (2011). An HNF4alpha-miRNA inflammatory feedback circuit regulates hepatocellular oncogenesis. Cell.

[81] Yang YM (2015). Galpha12 overexpressed in hepatocellular carcinoma reduces microRNA-122 expression via HNF4alpha inactivation, which causes c-Met induction. Oncotarget.

[82] Panda, M. & Biswal, B. K. Cell signaling and cancer: a mechanistic insight into drug resistance. *Mol. Biol. Rep*. 10.1007/s11033-019-04958-6 (2019). 10.1007/s11033-019-04958-6 31280421

[83] Suriyamurthy, S., Baker, D., Ten Dijke, P. & Iyengar, P. V. Epigenetic reprogramming of TGF-beta signaling in breast cancer. *Cancers (Basel)*, 10.3390/cancers11050726 (2019). 10.3390/cancers11050726 PMC6563130 31137748

[84] Gunewardena SS (2015). Deciphering the developmental dynamics of the mouse liver transcriptome. PLoS ONE.

[85] de Lucas S, Lopez-Alcorocho JM, Bartolome J, Carreno V (2004). Nitric oxide and TGF-beta1 inhibit HNF-4alpha function in HEPG2 cells. Biochem. Biophys. Res. Commun..

[86] Cozzolino AM (2013). TGFbeta overrides HNF4alpha tumor suppressing activity through GSK3beta inactivation: implication for hepatocellular carcinoma gene therapy. J. Hepatol..

[87] Santangelo L (2011). The stable repression of mesenchymal program is required for hepatocyte identity: a novel role for hepatocyte nuclear factor 4alpha. Hepatology.

[88] Cicchini C (2015). Epigenetic control of EMT/MET dynamics: HNF4alpha impacts DNMT3s through miRs-29. Biochim. Biophys. Acta.

[89] Kogure T (2014). Involvement of miRNA-29a in epigenetic regulation of transforming growth factor-beta-induced epithelial-mesenchymal transition in hepatocellular carcinoma. Hepatol. Res..

[90] Carmona FJ (2014). A comprehensive DNA methylation profile of epithelial-to-mesenchymal transition. Cancer Res.

[91] Saito Y (2003). Increased protein expression of DNA methyltransferase (DNMT) 1 is significantly correlated with the malignant potential and poor prognosis of human hepatocellular carcinomas. Int. J. Cancer.

[92] Yuan X (2009). Identification of an endogenous ligand bound to a native orphan nuclear receptor. PLoS ONE.

[93] Marchetti A, Bisceglia F, Cozzolino AM, Tripodi M (2015). New Tools for Molecular Therapy of Hepatocellular Carcinoma. Diseases.

[94] Serrels A (2006). Identification of potential biomarkers for measuring inhibition of Src kinase activity in colon cancer cells following treatment with dasatinib. Mol. Cancer Ther..

[95] Wei L (2017). Oroxylin A activates PKM1/HNF4 alpha to induce hepatoma differentiation and block cancer progression. Cell Death Dis..

[96] Song K (2013). Epigenetic regulation of MicroRNA-122 by peroxisome proliferator activated receptor-gamma and hepatitis b virus X protein in hepatocellular carcinoma cells. Hepatology.

[97] Kim JH (2019). Differential effects, on oncogenic pathway signalling, by derivatives of the HNF4 alpha inhibitor BI6015. Br. J. Cancer.

[98] Hu Q, Li L, Zou X, Xu L, Yi P (2018). Berberine attenuated proliferation, invasion and migration by targeting the AMPK/HNF4alpha/WNT5A pathway in gastric carcinoma. Front Pharm..

[99] Algamas-Dimantov A, Yehuda-Shnaidman E, Peri I, Schwartz B (2013). Epigenetic control of HNF-4alpha in colon carcinoma cells affects MUC4 expression and malignancy. Cell Oncol. (Dordr.).

[100] Ansari D (2014). Apicidin sensitizes pancreatic cancer cells to gemcitabine by epigenetically regulating MUC4 expression. Anticancer Res..

